# A Nomogram for Predicting Event-Free Survival in Childhood Acute Lymphoblastic Leukemia: A Multicenter Retrospective Study

**DOI:** 10.3389/fonc.2022.854798

**Published:** 2022-03-29

**Authors:** Yun-yan He, Xiao-jing Wu, Dun-hua Zhou, Li-hua Yang, Hui-rong Mai, Wu-qing Wan, Xue-qun Luo, Min-cui Zheng, Jun-lin Zhang, Zhong-lv Ye, Hui-qin Chen, Qi-wen Chen, Xing-jiang Long, Xiao-fei Sun, Ri-yang Liu, Qiao-ru Li, Bei-yan Wu, Li-na Wang, Xian-ling Kong, Guo-hua Chen, Xian-yan Tang, Jian-pei Fang, Ning Liao

**Affiliations:** ^1^Department of Pediatrics, The First Affiliated Hospital of Guangxi Medical University, Nanning, China; ^2^Graduate School, Guangxi Medical University, Nanning, China; ^3^Children’s Medical Center, Sun Yat-Sen Memorial Hospital, Sun Yat-Sen University, Guangzhou, China; ^4^Department of Pediatrics, Southern Medical University Zhujiang Hospital, Guangzhou, China; ^5^Department of Hematology and Oncology, Shenzhen Children’s Hospital, Shenzhen, China; ^6^Department of Pediatrics, Second Xiangya Hospital of Central South University, Changsha, China; ^7^Department of Pediatrics, Sun Yat-Sen University First Affiliated Hospital, Guangzhou, China; ^8^Department of Hematology, Hunan Children’s Hospital, Changsha, China; ^9^Department of Pediatrics, Affiliated Hospital of Guangdong Medical University, Zhanjiang, China; ^10^Department of Pediatrics, Third Affiliated Hospital of Sun Yat-Sen University, Guangzhou, China; ^11^Department of Pediatrics, First Affiliated Hospital of Nanchang University, Nanchang, China; ^12^Department of Pediatrics, Liuzhou People’s Hospital, Liuzhou, China; ^13^Department of Pediatrics, Sun Yat-Sen University Cancer Center, Guangzhou, China; ^14^Department of Pediatrics, Huizhou Central People’s Hospital, Huizhou, China; ^15^Department of Pediatrics, Zhongshan People’s Hospital, Zhongshan, China; ^16^Department of Pediatrics, The First Affiliated Hospital of Shantou University Medical College, Shantou, China; ^17^Department of Pediatrics, Guangzhou First People’s Hospital, Guangzhou, China; ^18^Department of Pediatrics, Boai Hospital of Zhongshan, Zhongshan, China; ^19^Department of Pediatrics, Huizhou First People’s Hospital, Huizhou, China; ^20^School of Public Health, Guangxi Medical University, Nanning, China

**Keywords:** childhood acute lymphoblastic leukemia, nomogram, prognostic factor, event-free survival, multicenter retrospective study

## Abstract

**Objective:**

Even though childhood acute lymphoblastic leukemia (ALL) has an encouraging survival rate in recent years, some patients are still at risk of relapse or even death. Therefore, we aimed to construct a nomogram to predict event-free survival (EFS) in patients with ALL.

**Method:**

Children with newly diagnosed ALL between October 2016 and July 2021 from 18 hospitals participating in the South China children’s leukemia Group (SCCLG) were recruited and randomly classified into two subsets in a 7:3 ratio (training set, n=1187; validation set, n=506). Least absolute shrinkage and selection operator (LASSO) and multivariate Cox regression analysis were adopted to screen independent prognostic factors. Then, a nomogram can be build based on these prognostic factors to predict 1-, 2-, and 3-year EFS. Concordance index (C-index), area under the curve (AUC), calibration curve, and decision curve analysis (DCA) were used to evaluate the performance and clinical utility of nomogram.

**Result:**

The parameters that predicted EFS were age at diagnosis, white blood cell at diagnosis, immunophenotype, ETV6-RUNX1/TEL-AML1 gene fusion, bone marrow remission at day 15, and minimal residual disease at day 15. The nomogram incorporated the six factors and provided C-index values of 0.811 [95% confidence interval (CI) = 0.792-0.830] and 0.797 (95% CI = 0.769-0.825) in the training and validation set, respectively. The calibration curve and AUC revealed that the nomogram had good ability to predict 1-, 2-, and 3-year EFS. DCA also indicated that our nomogram had good clinical utility. Kaplan–Meier analysis showed that EFS in the different risk groups stratified by the nomogram scores was significant differentiated.

**Conclusion:**

The nomogram for predicting EFS of children with ALL has good performance and clinical utility. The model could help clinical decision-making.

## Introduction

Acute lymphoblastic leukemia (ALL) is a malignant disease that mainly derived from B-lineage lymphoid progenitor cells or T-lineage lymphoid progenitor cells, which causes the proliferation, survival and maturation of leukemia cells, and finally leads to the lethal accumulation of leukemia cells (1, 2). Accumulation of leukemic cells leads to a decrease in the production of normal blood cells, resulting in a series of clinical manifestations. ALL is the most common type of childhood malignant. It accounts for about a quarter of all childhood neoplastic diseases and 80% of childhood leukemia (3, 4). With increasingly precise risk stratification, enhanced supportive care, and targeted therapy for specific fusion genotypes, 5-year event-free survival (EFS) rate for pediatric ALL can reach 85%, and 5-year overall survival (OS) rate is even higher than 90% (5, 6). Even though the overall treatment effect of ALL in children is gradually improving, about 20% of patients eventually relapse, resulting in lower survival rate and becoming a main reason for treatment failure, especially in those patients with high-risk factors (7).

Previous studies have shown that the prognosis of ALL is related to many factors, such as age at diagnosis (8–10), peripheral white blood cell (WBC) count at diagnosis (11–13), extramedullary leukemia status (14), immunophenotype, cytogenetic and molecular genetic characteristics (15–17), and therapeutic response (18). Early treatment response was considered to be an important prognostic factor for childhood ALL (19, 20). The assessment major includes the sensitivity response to the prednisone test on days 8 (D8-SRP), bone marrow remission (BMR) and minimal residual disease (MRD) level on days 15 and 33 in induction chemotherapy. These indicators reflect the clearance rate of leukemic cells during treatment and help to identify which patients are at high risk of relapse. Therefore, we can reassess the level of risk and adjust the intensity of treatment based on these indicators to improve patient outcomes.

As far as we know, few multicenter studies have conducted a comprehensive analysis of the overall predictive value of these influencing factors. Moreover, tumor heterogeneity and treatment response are easily overlooked. Therefore, a comprehensive and effective model is needed to identify high-risk patients earlier and accurately predict the patients’ survival. Nomogram has been proposed as a quantitative tool for risk and benefit assessment and make personalized precision medicine possible (21, 22). In recent years, the use of nomogram has become widespread. Nevertheless, nomogram for the estimation of children’s EFS with ALL have not yet been established. Hence, we aimed to construct an easily applicable nomogram to predict EFS in childhood ALL based on the South China children’s leukemia Group (SCCLG)-ALL-2016 multicenter study. By combining clinical characteristics and early treatment response, we can identify children at high-risk early and develop individualized treatment plans to improve prognosis.

## Materials and Methods

### Patients

**Figure 1:**
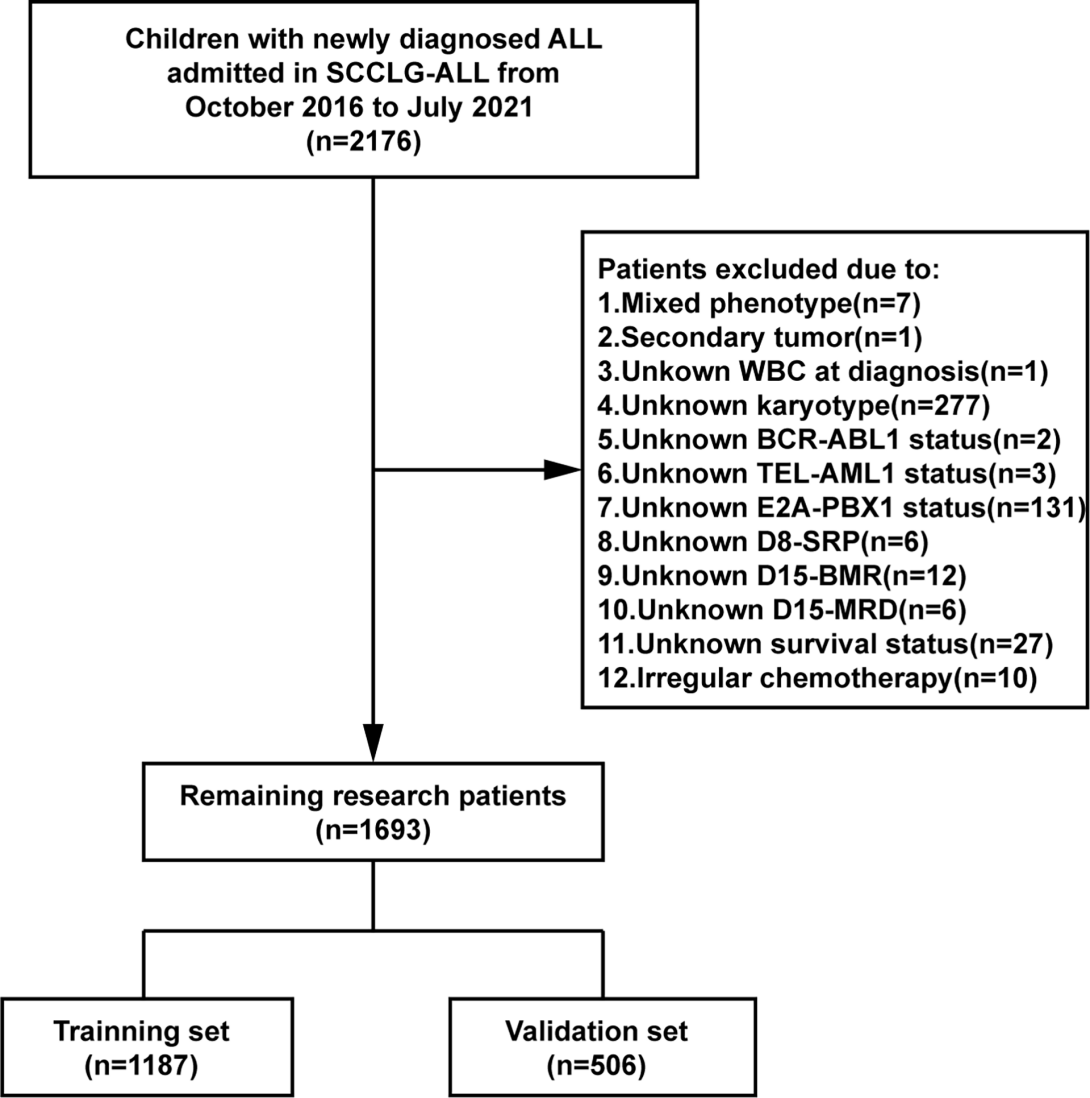
Flow chart of the study patients’ selection.

A multicenter retrospective study was conducted on 2176 newly diagnosed child with ALL from 18 hospitals participating in the SCCLG between October 2016 and July 2021. The inclusion criteria were: age ≤ 18 years, treated according to SCCLG-ALL-2016 protocol. The exclusion criteria were: mature B-ALL, mixed phenotype leukemia, definite chronic granulocytic leukemia acute, as a second tumor, secondary to immunodeficiency disease, with Down’s syndrome, non-primary, not treated according to protocol, incomplete data for the observed variables. After screening by inclusion and exclusion criteria, 1693 patients were eventually included for analysis and randomly classified into two subsets in a 7:3 ratio, namely the training set (n = 1187) and the validation set (n = 506). The detail screening process was shown in **Figure 1**. The study was reviewed and approved by the Ethics Committee of Sun Yat-sen Memorial Hospital, Sun Yat-sen University.

### Data Collection

The following information were collected: clinical characteristics including gender, age at diagnosis, peripheral WBC count at diagnosis, central nervous system (CNS) status, karyotype, immunophenotype, fusion gene status including *BCR-ABL1*, *MLL* rearrangements, *ETV6-RUNX1/TEL-AML1*, and *E2A-PBX1/TCF3-PBX1*, and early treatment response including D8-SRP of induction therapy, BMR at day 15 (D15-BMR) and day 33 (D33-BMR) of induction therapy, MRD determined by flow cytometry (FCM) at day 15 (D15-MRD) and day 33 (D33-MRD) of induction therapy, EFS events, and survival time.

For continuous variables, we converted them to categorical variables. Age was divided into three groups: < 1 year, 1**–**10 years, and ≥ 10 years at diagnosis (12). WBC was divided into two groups: < 100×10^9^/L and ≥ 100×10^9^/L at diagnosis (13). According to the risk stratification of SCCLG-ALL-2016 protocol, BMR and MRD were divided into three groups. D15-BMR and D33-BMR: bone marrow blasts < 5%, 5%**–**25%, ≥25%. D15-MRD: < 0.1%, 0.1%**–**10%, ≥ 10%. D33-MRD: < 0.01%, 0.01%**–**1%, ≥ 1%. CNS status was classified into three groups based on clinical manifestations, imaging (CT/MRI) findings, cerebrospinal fluid (CSF) WBC, red blood cell (RBC), and leukemia blasts. CNS1: no blast cells in the CSF and other signs of CNS leukemia. CNS2: CSF WBC ≤ 5/μl with blasts, or CSF WBC > 5/μl but the proportion of leukemia cells lower than that of peripheral blood naive cells (CSF WBC/RBC < 2×peripheral blood WBC/RBC) in traumatic lumbar puncture (TLP), or during LP, the peripheral blood WBC > 50×10^9^/L and accompanied by TLP. CNS3: CSF WBC > 5/μl with blasts in non-TLP, or CSF WBC > 5/μl and the proportion of leukemia cells higher than that of peripheral blood naive cells (CSF WBC/RBC ≥ 2× peripheral blood WBC/RBC) in TLP, or any clinical or imaging evidence of CNS leukemia regardless of CSF results. After seven days of oral prednisone according to the protocol, peripheral blood blasts <1.0×10^9^/L on day 8 was considered as prednisone good response (PGR), otherwise it was considered as prednisone poor response (PPR).

EFS was considered as outcome in this study. It was defined as the time from diagnosis to the first event or to the last follow-up, with events including induction failure (IF), relapse, and death. Failure to achieve complete remission (CR) at day 33 of induction chemotherapy was considered as IF. Hereon we would like to emphasize that CR was defined according to the latest recommendations by an international consensus of the Ponte-di-Legno Consortium: (1) MRD < 1% and/or BMR < 5%, (2) No extramedullary leukemia, (3) Pre-existing leukemic masses (including mediastinal masses) reduced to at least 1/3 of the initial tumor volume (23). The follow-up period for this study ended on July 31, 2021.

### Establishment and Validation of the Nomogram

The least absolute shrinkage and selection operator (LASSO) regression was performed to primarily screen risk factors for EFS in training set. This method can effectively avoid over-fitting in variable screening. Subsequently, significant prognostic factors screened by LASSO regression were further analyzed in multivariable Cox regression to identify independent prognostic factors associated with EFS of childhood ALL. Then, nomogram models for 1-year, 2-year, and 3-year EFS were constructed based on the variables with a *P* value<0.05 in multivariable Cox regression analysis.

We validated the nomogram both internally (training set) and externally (validation set) to evaluate its performance. The discrimination ability of nomogram was assessed by concordance index (C-index) and the area under the ROC (receiver operating characteristic) curve (AUC). The closer the C-index and AUC value are to 1, the better the model performance is. Generally, C-index > 0.75 represents relatively good discrimination (24). While AUC values of ≥ 0.90, 0.80–0.89, 0.70–0.79, and < 0.70 were considered as outstanding, excellent, acceptable, and poor discrimination, respectively (25). The degree of conformity between the predicted prognosis of nomogram and the actual prognosis is evaluated by the calibration curve (1000 bootstrap resamples). The closer the calibration curves are to the middle diagonal line, the better the prediction accuracy of this nomogram. Finally, the clinical usefulness of nomogram model was estimated by decision curve analysis (DCA). The larger the area under the curve, the better the clinical utility.

In addition, we established a risk stratification system based on the total scores calculated from the nomogram. Kaplan–Meier curves and the log-rank test were performed to compare the EFS of childhood ALL patients in different groups.

### Statistical Analysis

SPSS 25.0 software (IBM, USA) and R software version 4.0.2 (Vienna, Austria; https://www.R-project.org) were used for statistical analysis. For comparisons between groups, the student’s t test was used for continuous variables, while the chi-square test was used for categorical variables. A *P*-value<0.05 means statistically significant. LASSO regression, multivariable Cox regression, nomogram, C-index, AUC, calibration curves, and DCA were conducted using R statistical packages “glmnet”, “rms”, “survival”, “timeROC”, “foreign”, “Hmisc”, and “ggDCA”.

## Results

### Patients Characteristics

The clinical characteristics and early treatment response of patients in training and validation set is shown in **Table 1**. A total of 213 EFS events were observed: 104 patients had IF, 58 relapsed patients, and 51 dead patients. The median of EFS was 744 days (range = 28**–**1754) in the training set and 639.5 days (range = 29**–**1757) in the validation set, respectively. There was no statistical difference in the observed variables between the training set and validation set (*P* > 0.05).

**Table 1:** Basic characteristics and EFS status of the patients.

Characteristics	Total (n = 1693)	Training set (n = 1187)	Validation set (n = 506)	*P* value
Gender				
Male	991 (58.5%)	706 (59.5%)	285 (56.3%)	0.228
Female	702 (41.5%)	481 (40.5%)	221 (43.7%)	
Age, years				
<1	16 (0.9%)	13 (1.1%)	3 (0.6%)	0.238
1-10	1415 (83.6%)	1000 (84.2%)	415 (82.0%)	
≥10	262 (15.5%)	174 (14.7%)	88 (17.4%)	
WBC (10^9^/L)				
<100	1462 (86.4%)	1019 (85.8%)	443 (87.5%)	0.350
≥100	231 (13.6%)	168 (14.2%)	63 (12.5%)	
CNS status				
CNS1	1604 (94.7%)	1126 (94.9%)	478 (94.5%)	0.272
CNS2	37 (2.2%)	22 (1.8%)	15 (3.0%)	
CNS3	52 (3.1%)	39 (3.3%)	13 (2.5%)	
Immunophenotype				
B-ALL	1504 (88.8%)	1052 (88.6%)	452 (89.3%)	0.675
T-ALL	189 (11.2%)	135 (11.4%)	54 (10.7%)	
Karyotype				
>44 chromosomes	1679 (99.2%)	1179 (99.3%)	500 (98.8%)	0.287
≤44 chromosomes	14 (0.8%)	8 (0.7%)	6 (1.2%)	
BCR-ABL1 status				
Negative	1593 (94.1%)	1111 (93.6%)	482 (95.3%)	0.185
Positive	100 (5.9%)	76 (6.4%)	24 (4.7%)	
MLL status				
Negative	1643 (97.0%)	1150 (96.9%)	493 (97.4%)	0.542
Positive	50 (3.0%)	37 (3.1%)	13 (2.6%)	
ETV6-RUNX1/TELAML1				
Negative	1389 (82.0%)	976 (82.2%)	413 (81.6%)	0.767
Positive	304 (18.0%)	211 (17.8%)	93 (18.4%)	
E2A-PBX1/TCF3-PBX1				
Negative	1608 (95.0%)	1125 (94.8%)	483 (95.5%)	0.559
Positive	85 (5.0%)	62 (5.2%)	23 (4.5%)	
D8-SRP				
PGR	1503 (88.8%)	1047 (88.2%)	456 (90.1%)	0.254
PPR	190 (11.2%)	140 (11.8%)	50 (9.9%)	
D15-BMR				
<5%	1390 (82.1%)	985 (83.0%)	405 (80.0%)	0.246
5%-25%	170 (10.0%)	110 (9.3%)	60 (11.9%)	
≥25%	133 (7.9%)	92 (7.7%)	41 (8.1%)	
D15-MRD				
<0.1%	666 (39.3%)	466 (39.3%)	200 (39.5%)	0.715
0.1%-10%	756 (44.7%)	536 (45.1%)	220 (43.5%)	
≥10%	271 (16.0%)	185 (15.6%)	86 (17.0%)	
EFS events				
Induction failure (IF)	104 (6.1%)	69 (5.8%)	35 (6.9%)	0.657
Relapse	58 (3.4%)	37 (3.1%)	21 (4.2%)	
Dead	51 (3.0%)	30 (2.5%)	21 (4.2%)	
EFS time (days)				
Median	724	744	639.5	
Range	28-1757	28-1754	29-1757	

WBC, white blood cell counts at diagnosis; CNS status, central nervous system status at diagnosis; MLL, mix lineage leukemia; D8-SRP, sensitivity response to the prednisone test on day 8 in induction chemotherapy; PGR, prednisone good response; PPR, prednisone poor response; D15-BMR, bone marrow remission on days 15 in induction chemotherapy; Day15-MRD, minimal residual disease level on days 15 in induction chemotherapy; EFS time (days), event-free survival time in days.

### Prognostic Factors for EFS

**Figure 2:**
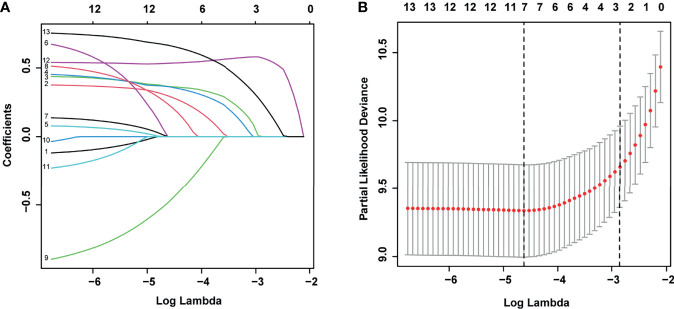
Least absolute shrinkage and selection operator (LASSO) regression for selecting prognostic factors associated with event-free survival (EFS). **(A)** LASSO coefficients of the 13 variables. **(B)** The selection of tuning parameter (λ) for LASSO *via* 10-fold cross-validation.

According to the LASSO regression analysis, seven risk factors were selected in the training set, including age and WBC at diagnosis, phenotype, *MLL* status, *ETV6-RUNX1/TEL-AML1* fusion status, D15-BMR, and D15-MRD (**Figure 2**). Through further multivariate Cox regression analysis (**Table 2**), six independent predictors of EFS were screened out in the training set, including age at diagnosis, WBC at diagnosis, phenotype, *ETV6-RUNX1/TEL-AML1* fusion status, D15-BMR, and D15-MRD.

**Table 2:** Multivariate Cox regression analysis based on the results of lasso regression.

Characteristics	HR	Lower 95%CI	Upper 95%CI	*P* value
Age, years				
<1	Reference			
1-10	0.24	0.07	0.79	0.019
≥10	0.44	0.13	1.50	0.185
WBC (10^9^/L)				
<100	Reference			
≥100	1.60	1.10	2.40	0.023
Immunophenotype				
B-ALL	Reference			
T-ALL	1.50	1.00	2.30	0.049
MLL status				
Negative	Reference			
Positive	1.60	0.76	3.30	0.223
ETV6-RUNX1/TELAML1				
Negative	Reference			
Positive	0.41	0.18	0.95	0.037
D15-BMR				
<5%	Reference			
5%-25%	1.60	0.90	2.70	0.112
≥25%	2.90	1.70	5.10	<0.001
D15-MRD				
<0.1%	Reference			
0.1%-10%	2.00	1.20	3.40	0.009
≥10%	5.10	2.60	9.70	<0.001

WBC, white blood cell counts at diagnosis; MLL, mix lineage leukemia; D15-BMR, bone marrow remission on days 15 in induction chemotherapy; Day15-MRD, minimal residual disease level on days 15 in induction chemotherapy; HR, hazard ratio; CI, confidence interval.

### Establishment and Validation of EFS Nomogram

**Figure 3:**
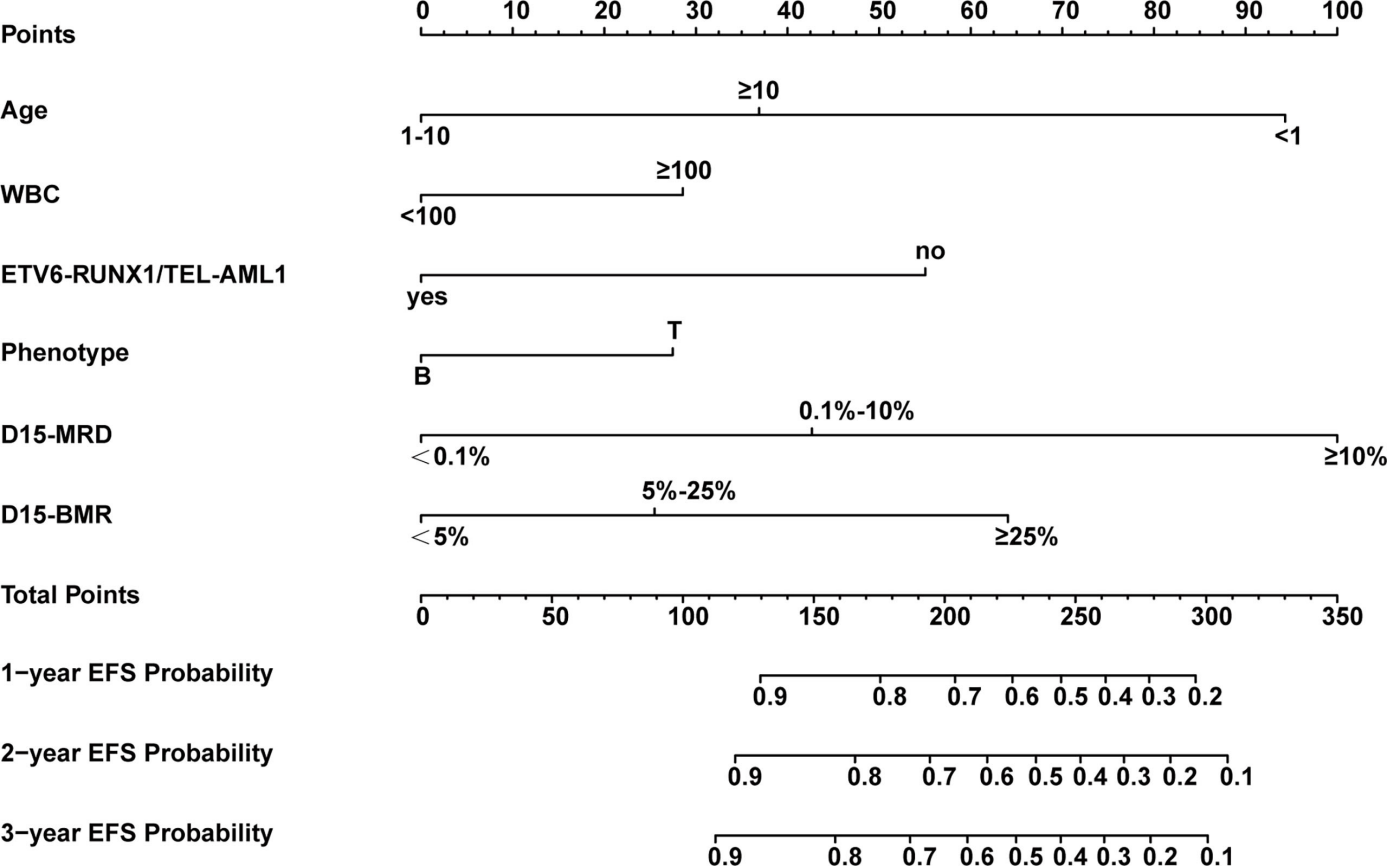
Nomogram model for predicting 1-, 2-, and 3-year event-free survival (EFS) probabilities in children with acute lymphoblastic leukemia (ALL).

The above six independent predictors were used to construct a nomogram to predict EFS in patients with childhood ALL (**Figure 3**). Each predictor has a single score corresponding to different values. The points of these six predictors were added to get the total score. The vertical corresponds to the probability of 1-, 2-, and 3-year EFS of each patient.

**Figure 4:**
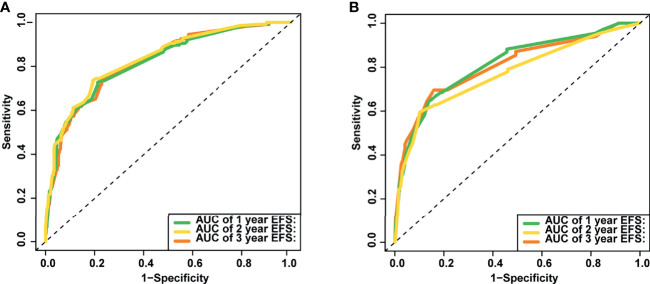
The area under the receiver operating characteristic (ROC) curves (AUCs) of the nomogram for predicting 1-, 2- and 3-year event-free survival (EFS) in training **(A)** and validation set **(B)**.

**Figure 5:**
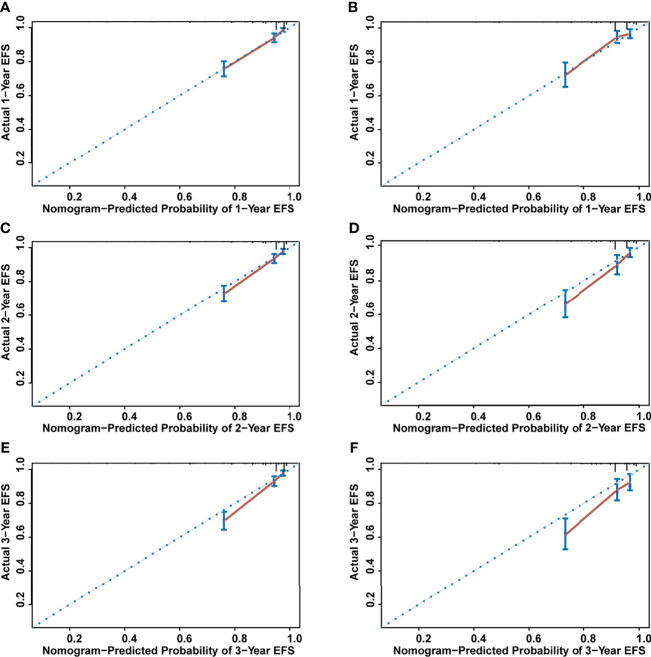
The calibration curves for assessing the calibration ability of the nomogram. **(A)** One-year event-free survival (EFS) for training set. **(B)** One-year EFS for validation set. **(C)** Two-year EFS for training set. **(D)** Two-year EFS for validation set. **(E)** Three-year EFS for training set. **(F)** Three -year EFS for validation set.

The nomogram’s performance was evaluated: the C-index were 0.811 (95% confidence interval (CI) = 0.792**–**0.830) and 0.797 (95% CI = 0.769**–**0.825) in the training and validation set, respectively. The ROC curve is shown in **Figure 4**. The AUC values for predicting 1-, 2-, and 3-year EFS in the training set were 0.822, 0.822, and 0.821, respectively, while in the validation set were 0.808, 0.808, and 0.814, respectively. Both C-index and AUC values exhibited a good discriminative ability of the nomogram. In addition, the calibration curves displayed that the 1-, 2-, and 3-year EFS probabilities predicted by the nomogram were in good agreement with the actual probabilities in both the training and validation sets (**Figure 5**).

**Figure 6:**
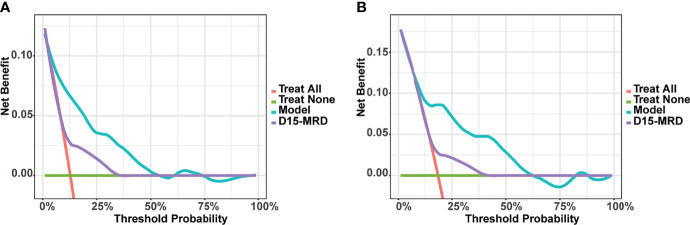
Decision curve analysis (DCA) of the nomogram model for predicting event-free survival (EFS) in training **(A)** and validation set **(B)**.

The DCA for predicting EFS in childhood ALL patients in this study is shown in **Figure 6**. The X-axis of DCA represents “threshold probability” and the Y-axis means the net benefit (NB). Compared with “Treat all”, “Treat none”, and “D15-MRD”, using the nomogram model to decide whether to intervene the treatment had a higher benefit, indicating that the nomogram in this study has good potential for clinical application.

### Risk Stratification System

**Figure 7:**
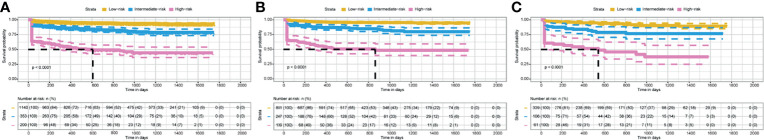
Kaplan–Meier curves of EFS for patients in different risk groups stratified by the nomogram scores. **(A)** In the entire study set. **(B)** In the training set. **(C)** In the validation set.

Based on the total model prediction scores, we stratified patients into three risk strata (low risk: score 0-109, intermediate risk: score 110-182, and high risk: score 183-312). Kaplan-Meier analysis showed that in the entire study set, the 3-year EFS rates in the above three groups were 94%, 80%, and 44%, respectively (*P*<0.001, **Figure 7A**). Similar results were observed in the training set and validation set (both *P*<0.001, **Figures 7B, C**).

## Discussion

The prognosis of ALL in children is influenced by many factors. These factors may be interrelated and affect each other. Therefore, how to comprehensively analyze these factors so that it can predict the ALL prognosis more accurately, intuitively, conveniently, and comprehensively in clinical applications is the focus of the present study. Based on SCCLG-ALL-2016 multicenter study, a nomogram incorporating age at diagnosis, WBC at diagnosis, phenotype, *ETV6-RUNX1/TEL-AML1* fusion status, D15-BMR, and D15-MRD was constructed by LASSO and multivariate Cox analysis to predict EFS probability in children with ALL. The nomogram model showed good discrimination and calibration in both the training and validation sets. Moreover, the results of DCA analysis showed that the nomogram model has good clinical application prospects. This nomogram is easy to use and provides an important basis for physicians to develop individualized treatment strategies for childhood ALL patients with predicted poor prognosis.

It’s worth noting that the rate of *BCR-ABL1* positive in this study was 5.9%, which is higher than the 3%-5% reported in previous study (26). We considered that this may be related to the following reasons: 1. In this study, fluorescent *in situ* hybridization (FISH) and polymerase chain reaction (PCR) were used to detect *BCR-ABL1* fusion, which may improve the positive detection rate. 2. Actually, the incidence of *BCR-ABL1* positive ALL in this study was close to another report from the South China Children’s Leukemia Group (27), suggesting that the positive rate of *BCR-ABL1* in the population of South China may just be higher than that in the regions involved in other protocols. In addition, the incidence of induction failure (IF) was higher than that reported in other protocols (28). Hereon, we would like to explain that the traditional definition of IF was bone marrow blasts ≥ 5% and/or other evidence of extramedullary leukemia after 4-6 weeks of remission-induction therapy, whereas according to the latest recommendation of an international consensus of the Ponte-di-Legno Consortium (23), D33-MRD ≥ 1% was also considered as a basis for IF. Hence, the rate of IF in this study was higher than that in most previous studies.

Among the reported factors affecting the prognosis of childhood ALL, MRD has been considered as a highly reliable prognostic indicator and a major cause of relapse (29, 30). It reflects both drug sensitivity and host pharmacodynamics, pharmacogenomics, treatment adherence, and efficacy. Monitoring of MRD levels can not only assess early treatment response, risk stratification, and treatment intensity stratification, but also determine prognosis, predict relapse, and guide hematopoietic stem cell transplantation, relapse ALL treatment, and individualized treatment (31–36). Basso G et al. (37) reported that the MRD at day 15 measurement by FCM was the strongest early predictor of relapse and is applicable to almost all patients. Jeha S et al. (38) found that the measurement of D15-MRD levels in bone marrow helped to identify patients with early adverse reactions. Treatment intensification for D15-MRD ≥ 1% improved outcome of patients who are in intermediate or unfavorable subtypes. O’Connor D et al. (39) found that patients with morphological remission of bone marrow but high MRD (≥ 5%) at the end of induction (EOI) had a similar prognosis to those who fail to achieve morphological remission, suggesting that MRD was more objective and accurate for disease response assessment compared to morphology. Consistent with previous studies, D15-MRD was found to be the most significant factor for poor prognosis in this study. It had the highest score in our nomogram model (score = 100 when MRD ≥ 10%), its predictive power was stronger than D15-BMR and other predictors, which confirmed the reliability of MRD again.

Previous studies have also shown that age at diagnosis was associated with survival prognosis in childhood ALL patients. Patients aged < 1 year and ≥ 10 years at initial diagnosis were reported to have a poor outcome than patients aged 1-10 years (9, 12). Our result was consistent with these aforementioned studies, and the prognosis was the worst for patients under one year old, as shown in the nomogram. High peripheral blood WBC count at diagnosis was also considered as a risk factor for childhood ALL, especially associated with the relapse of B-ALL (11–13). This study also demonstrated that the EFS experience for patients with WBC ≥ 100×10^9^/L was worse than that for patients with WBC < 100×10^9^/L. Moreover, research in recent years have shown that patients with central nervous system leukemia (CNSL), T-cell immunophenotype, low hypodiploid, *BCR-ABL1* positive, *E2A-PBX1/TCF3-PBX1* positive, or *MLL* rearrangements also had a poor prognosis (14–17). Conversely, patients with *ETV6-RUNX1/TEL-AML1* positive had a better prognosis (27, 40). In the present study, we found that T-cell immunophenotype was a risk factor for EFS, while *ETV6-RUNX1/TEL-AML1* was a protective factor, which were agreement with previous reports. The contribution rates of risk factors in the nomogram are represented by the length of line segments, and the score of these factors represents the degree of their influence on EFS. However, CNSL, hypodiploid (≤ 44 chromosomes), *BCR*-*ABL1* gene fusion, *E2A*-*PBX1*/*TCF3*-*PBX1* gene fusion, and MLL rearrangements were not identified significantly in this nomogram. This may be related to the following reasons: 1) The proportion of low hypodiploid (≤ 44 chromosomes) in this study population was very low (0.8%) and may not be well represented, so further data collection is needed to increase the sample size. 2) Although other factors such as *BCR-ABL1* positivity rate was not low, they may interact with each other and have less significant effect on prognosis than the single factor study, so the Nomogram model was not included. 3) The optimal cut-off values of research variables were not uniform, resulting in different results. 4) The follow-up period of this study was not long enough, and the endpoint event in most previous studies was OS, whereas this study aimed to assess EFS. Of course, statistical significance cannot fully represent clinical significance. Although we could include these factors in the nomogram from a clinical perspective, this would make nomogram too complicated, and we found that it would reduce the efficiency of the model and make it difficult for clinical use. In contrast, the prediction model established after screening variables by LASSO and multivariate Cox regression analysis has the advantages of good performance, fewer variables, and convenient clinical use. At the same time, the overfitting of the model was avoided, and the clinical applicability and accuracy of the model were improved.

There are several advantages of our study. Firstly, compared to the other two previous studies (41, 42), this study is the first to use the SCCLG-ALL-2016 multicenter study with a large sample size to establish EFS nomogram. This is more applicable to assess the prognosis of Chinese childhood ALL patients. Secondly, most of the previous studies were limited to single risk factor and lacked a comprehensive analysis of multiple prognostic factors. However, in our clinical work, we found that a patient may actually had several risk factors at the same time, or risk factors and protective factors coexist. It is a challenge to develop a more accurate risk stratification and treatment plan for such patients. In the present study, we established an EFS nomogram combining clinical characteristics, cytogenetics, molecular genetics, and early treatment response, which provides a more comprehensive assessment of the patient’s risk level. The factors in the nomogram are objective and easy to obtain, and the DCA curve showed that its predictive value was significantly better than that predicted by each factor independently such as D15-MRD, which is important for guiding individualized treatment. Overtreatment can be avoided in children at low-risk, while children at high-risk require more intensive treatment. For example, a 3-year-old male patient newly diagnosed with B-ALL was admitted with WBC 120×10^9^/L, *ETV6-RUNX1* negative, D-15 BMR 8%, and D15-MRD 12%. According to the EFS nomogram, the patient was scored with 209 points (29 + 55+25+100) and was in the high-risk group. Clinicians can be guided to formulate more intensive chemotherapy and prepare for transplantation as early as possible. If the patient had a positive *ETV6-RUNX1* (a protective factor), he would have received a score of 154 points (29 + 25+100) and be considered at moderate risk. This means he doesn’t need a treatment as strong as the former. However, these results need to be further validated in the clinic, especially with prospective studies.

Meanwhile, this study also has some limitations. First, it was a retrospective study, and part of patients were excluded because of incomplete information, which may lead to selection bias. Second, some potential prognostic predictors such as intrachromosomal amplification of chromosome 21 (iAMP21) and Ikarus plus (i.e. *IKZF1* deletion in concert with *CDKN2A/B*, *PAX5* or *PAR1* deletion) were not included in the discussion. This study is a multi-center study, and these centers are located in different regions with different economic conditions. Some large and expensive tests such as panoramic gene sequencing cannot be fully implemented, so these factors were not investigated in the present study. It is expected that those data will be refined in the future, as a more comprehensive model may have better prognostic predictive performance. Third, the follow-up period in this study was not long enough, and these patients still need close monitoring and followed-up. Finally, this study only conducted internal validation, we need collect data for external validation in the future to further evaluate the value of the model.

In conclusion, we comprehensively evaluated the factors related to the prognosis of children with ALL, and constructed a nomogram that can objectively and accurately predict the EFS for childhood ALL patients. Adequate and rational use of nomogram can help clinicians to formulate individualized treatment plans, thereby improving the survival rate of children with ALL.

## Data Availability Statement

The raw data supporting the conclusions of this article will be made available by the authors, without undue reservation.

## Ethics Statement

The studies involving human participants were reviewed and approved by Ethics Committee of Sun Yat-Sen Memorial Hospital, Sun Yat-Sen University. Written informed consent to participate in this study was provided by the participants’ legal guardian/next of kin.

## Author Contributions

Y-yH and X-jW designed the study. Y-yH, NL, D-hZ, J-pF, L-hY, H-rM, W-qW, X-qL, M-cZ, Z-lY, H-qC, Q-wC, X-jL, X-fS, R-yL, Q-rL, B-yW, L-nW, X-lK, and G-hC obtained and assembled data. Y-yH, X-jW, and J-lZ analyzed and interpreted the data. X-jW wrote the manuscript. X-jW, J-lZ, and X-yT did the statistical analysis. All authors reviewed the manuscript and approved the final version.

## Conflict of Interest

The authors declare that the research was conducted in the absence of any commercial or financial relationships that could be construed as a potential conflict of interest.

## Publisher’s Note

All claims expressed in this article are solely those of the authors and do not necessarily represent those of their affiliated organizations, or those of the publisher, the editors and the reviewers. Any product that may be evaluated in this article, or claim that may be made by its manufacturer, is not guaranteed or endorsed by the publisher.
